# Evaluation of different blood-feeding frequencies on *Glossina palpalis gambiensis* performance in a mass-rearing insectary

**DOI:** 10.1186/s13071-020-04559-4

**Published:** 2021-01-13

**Authors:** Karifa Camara, Kadidiata Ilboudo, Ernest Wendemanegde Salou, Geoffrey Gimonneau

**Affiliations:** 1 Centre International de Recherche–Développement sur l’Elevage en zone subhumide (CIRDES), BP 454, Bobo-Dioulasso 01, Burkina Faso; 2 Institut Supérieur des Sciences et de Médecine Vétérinaire (ISSMV), BP 09, Dalaba, Guinea; 3 Département de Sciences biologiques/UFR-ST, Université́ Nazi Boni (UNB), BP 1091, Bobo-Dioulasso, Burkina Faso; 4 INTERTRYP, Unité mixte de recherche (UMR), Centre de coopération internationale en recherche agronomique pour le développement (CIRAD), 34398 Montpellier, France; 5 INTERTRYP, Institut de Recherche pour le Développement (IRD), CIRAD, Université de Montpellier, Montpellier, France

**Keywords:** Tsetse fly, Productivity, Survival, Emergence rate, Flight ability

## Abstract

**Background:**

The main challenge to the successful mass-rearing of the tsetse fly in insectaries, especially in Africa, is a sustainable supply of high-quality blood meals. As such, the collection of high-quality blood in large quantities can be an important constraint to production. One possible strategy to lessen the impact of this constraint is to modify the blood-feeding frequency. In the study reported here, we evaluated the effect of three blood-feeding frequencies on the colony performance of* Glossina palpalis gambiensis*, a riverine tsetse fly species.

**Methods:**

The effect of three, four and six blood-feedings per week on female survival and productivity were evaluated over a 30-day period. Progeny emergence rate and flight ability were also evaluated.

**Results:**

Female survival was significantly higher in flies fed four times per week (87%) than in those fed three (72%) and six times per week (78%;* P* < 0.05). Productivity was similar between flies fed four and six times per week (457 and 454 larvae) but significantly reduced in flies fed three times per week (280 larvae produced;* P* < 0.05). Both emergence rate and flight ability rate were also similar between flies fed four times per week (97 and 94%, respectively) and six times per week (96 and 97%, respectively), but they were significantly reduced when flies were fed three times per week (89 and 84%, respectively;* P* < 0.05).

**Conclusions:**

Blood-feeding frequency could be reduced from six times per week to four times per week without affecting mass-rearing production and progeny quality. The implications of these results on tsetse mass-rearing production are discussed.
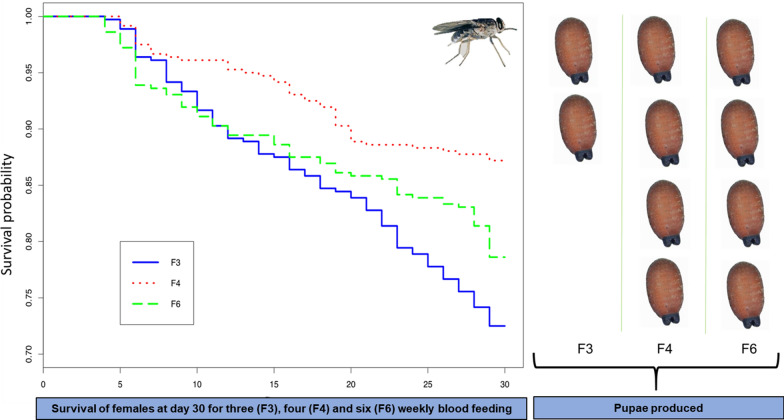

## Background

Tsetse flies (Diptera: Glossinidae) are the cyclical vector of African trypanosomes across sub-Saharan Africa which cause human African trypanosomosis (HAT) and African animal trypanosomosis (AAT) two debilitating diseases of humans (sleeping sickness) and livestock (nagana), respectively [1, 2]. Due to their distribution across 36 countries in sub-Saharan Africa, tsetse flies impair the development of sustainable and productive agricultural systems in more than ten million km^2^ in the region [3, 4], leading to potential losses in livestock and crop production that are estimated to be USD 4.75 billion per year [5]. To date, animal trypanosomosis is mainly controlled through prophylactic and curative drugs, but this approach is no longer sustainable due to the development of drug resistance [6, 7]. Therefore, the current method to protect people and livestock efficiently is to reduce host vector contact through vector control of sibling species [8].

Vector control programs are based as much as possible on an area-wide integrated pest management (AW-IPM) approach and depending on the environmental context may integrate the sterile insect technique (SIT) component [9, 10]. SIT is a species-specific and environmentally friendly biologically based control tactic to manage populations (suppression and/or elimination) of insect pests and disease vectors [11]. The technique consists of the mass production of sterilized male adults that are released in the field with the aim to out-compete wild males and mate with wild virgin females. This mating is not productive and will lead to population reduction or elimination [12]. The SIT has been used successfully in the past for the eradication of tetse fly* Glossina austeni* in Unguja Island, Zanzibar [13] and is currently being used in the Niayes area of Senegal to control *Glossina palpalis gambiensis* [14].

The tsetse flies released in Senegal are provided to the eradication program as chilled irradiated male pupae by the Centre International de Recherche-Développement sur l’Elevage en zone Subhumide (CIRDES) based in Bobo-Dioulasso, Burkina Faso [14]. CIRDES hosts a mass-rearing insectary with the largest colony of *G. p. gambiensis* in Africa. In tsetse flies, both sexes are obligate blood-feeders, and one of the main challenges in successfully managing tsetse fly colonies is to maintain a high production rate while reducing production costs and producing pupae of good quality. These results can be achieved by using high-quality blood to feed tsetse colonies and through a regular assessment of colony performance through measurement of the colony’s biological parameters, such as longevity, fecundity and pupae quality. One way to reduce production costs is to optimize colony feeding frequency because as the frequency of feeding increases, so does the production cost of flies (staff and blood collection). Moreover, too many manipulations of the flies, such as regular exposure to heating plates or transfer from one room to another, can be detrimental to colony production and also increase the risk of the transmission of microorganisms due to multiple feeding [15]. In this context, the objective of this study was to evaluate different feeding frequencies on *G. p. gambiensis* performance in the setting of mass-rearing production. Three feeding frequencies were evaluated, namely three, four and six times per week, and four biological parameters were measured: (i) female survival, (ii) productivity, (iii) progeny emergence rate and (iv) flight ability. These parameters were used to assess the effect of feeding frequency on fly performance.

## Methods

### Insectary

The study was carried out at the CIRDES insectary in Bobo-Dioulasso, Burkina Faso. This is a mass-rearing insectary for *G. p. gambiensis* set up in 1975 to support the national and regional eradication of tsetse flies [16]. Approximately 250,000 females are reared, which produce more than 30,000 male pupae per week. The pupae are currently being shipped as chilled irradiated pupae for release in the eradication program being conducted in the Niayes area of Senegal [14]. Tsetse flies in the mass-rearing facility are maintained at 24–25 °C, 75–80% relative humidity and under a photoperiod of 5:19 h (light:dark).

### Biological material

Teneral flies of the *G. p. gambiensis* colony were used in this study. This tsetse colony was established in 1972 at Maison-Alfort (France) with pupae collected from Guinguette (Bobo-Dioulasso, Burkina Faso). In 1975 the colony was transferred to the Centre de Recherche sur la Trypanosomiase Animale (CRTA) (later renamed CIRDES), consisting of 5333 pupae [16]. In 1981, the colony was replenished with wild material from the ‘Marre aux hippopotames’ [17]. Flies are maintained on fresh irradiated bovine blood collected from the local slaughterhouse using an* in vitro* silicon membrane system [18]. The standard feeding frequency is six times per week, from Monday to Saturday.

### Blood collection and conditioning

Blood from different animals (cattle and pigs) was collected at the Bobo-Dioulasso slaughterhouse in 10-L sterile containers and defibrinated using a custom-made stainless steel electric paddle stirrer. On arrival at the insectary, glucose (diluted in 10 mL distilled water) was added to the blood (1 g/L blood) as a phagostimulant [19], following which the blood was irradiated with 500 Gy from a ^137^Cs source for 1 h and 40 min and stored in 2-L containers at 4 °C. After irradiation, the blood was tested for bacterial contamination by inoculating 1 mL of a blood sample with a syringe onto agar-filled Petri dishes and incubating for 72 h. After incubation, if the irradiated blood contained no more than ten bacterial colonies, it was kept for feeding; it was discarded if more than ten colonies were counted. All procedures involving the handling of blood were performed under a laminar flow hood to avoid bacterial contamination.

### Experimental procedure

The effect of blood-feeding frequency was evaluated using measurements of several biological parameters that are routinely checked in tsetse colonies as indicators of colony performance. Female survival (percentage alive on day 30), productivity (number of pupae produced at day 30), fecundity, first larviposition date, pupal size, pupal emergence rate and flight ability of newly emerged flies were assessed for each blood-feeding treatment. Among these, female survival, fecundity and pupal size were used in a formula to calculate a quality factor (QF) of the blood-feeding frequency as a comprehensive indicator of colony production [20, 21]. The detailed formula is described in De Beer et al. [22]. A QF of > 1 indicates that blood is suitable for colony maintenance.

To evaluate the effect of blood-feeding frequency, three experimental treatments were performed. Flies were fed three times per week (F3) on Monday, Wednesday and Friday; four times per week (F4) on Monday, Tuesday, Thursday and Friday; or six times per week (F6) from Monday to Saturday. Newly emerged flies were blood fed using an* in vitro* silicon membrane feeding system with the appropriate blood treatment over the course of 30 days. Bioassays were conducted using ten 6-day-old males and 30 3-day-old females per cage. Females were monitored daily for survival and productivity (pupal production and abortion). The pupae produced were sorted in five class sizes (A–E) calibrated for *G. p. gambiensis* according to their weight: A (< 22 mg), B (22 to < 28 mg), C (28 to < 32 mg), D (32 to < 36 mg) and E (> 36 mg) [21]. After 30 days, all surviving females were dissected to determine their reproductive status (presence/absence of egg/larvae in the uterus and insemination status). For each feeding frequency, the pupae produced were put in Petri dishes under approximately 1 cm of sand and covered with a flight cylinder [14]. The inner wall of the cylinder was coated with unscented talcum powder to prevent the flies from crawling out. This method was used to assess the number of flies able to fly out, which are classified as operational flies for the SIT approach. After emergence, the number of pupae that did not emerge was counted. This study was performed between January and June 2018, and four cohorts of flies were studied for all treatments. For each cohort, all bioassays were replicated three times, leading overall to 12 replicates per treatment.

### Statistical analyses

The survival of female flies fed at different feeding frequencies was analyzed using Kaplan–Meier survival curves. Survival curves were compared using the ‘coxme’ function where the blood treatment was used as an explanatory variable, survival was used as the response variable and cohorts and replicates were used as random effects.

Female productivity, first larviposition date and pupal classes were analyzed using a generalized linear mixed effects model with a ‘Poisson’ family. Quality factors were tested using linear mixed effects models. The adult emergence rate and percentage of flies able to fly were analyzed using binomial generalized mixed effects models. For each model, the treatments were used as a fixed effect and cohorts were used as a random effect. The best model was selected on the basis of the lowest corrected Akaike information criterion, and the significance of fixed effect was tested using the likelihood test ratio [23, 24]. R software (version 3.5.0) was used for data analysis [25].

## Results

### Survival

**Fig. 1:**
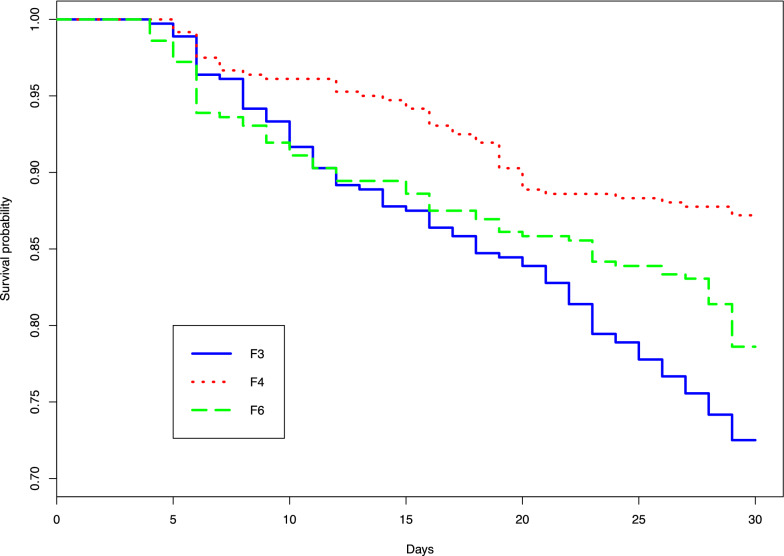
Survival curves of flies fed at different frequencies per week.* F3* three blood meals per week,* F4* four blood meals per week,* F6* six blood meals per week

A total of 360 females were blood-fed for 30 days for each feeding frequency: three days per week (F3), four days per week (F4) and six days per week (F6). On day 30, survival was highest for females fed four times per week (87.2%) and lowest for flies fed three times (72.5%, Table 1, Fig. 1). Model analysis showed that the frequency of blood-feeding had a significant effect on female survival (likelihood-ratio test:* χ*^2^ = 54.171,* df* = 2, *P* > 0.001). The survival rate of females fed according to treatment F3 was significantly lower than that of flies fed according to treatments F4 (*P <* 0.001) and F6 (*P* = 0.047). However, the survival of females in treatment F4 was significantly higher than that for females in treatment F6 (*P* = 0.003).

**Table 1:** Effect of different blood feeding frequencies on *Glossina palpalis gambiensis* survival, productivity and fertility

Blood-feeding treatment^a^	No. of females surviving at day 30 (%)	No.of mature females		No. of pupae produced	Pupae size classes^b^			Fecundity	Quality factor^c^	Uterus					Insemination rate	Pupae quality control	
										No. of recently ovulated egg (%)	No. of empty eggs due to abortion (%)	No. of viable instar larvae					
		Day 18	Day 30		A	B	C					First (%)	Second (%)	Third (%)		No. of emergences (%)	No. of operational flies (%)
F3	261 (72.5)	1352	1325	280	99	174	6	0.072	1.18	28 (24.4)	21 (18.3)	23 (20)	30 (26)	13 (11.3)	1	250 (89.3)	210 (84)
F4	314 (87.2)	1394	1378	457	126	314	17	0.092	1.48	24 (8.8)	14 (5.1)	85 (31.1)	98 (35.9)	52 (19.1)	1	445 (97.4)	419 (94.15)
F6	283 (78.6)	1363	1333	454	112	301	41	0.087	1.59	8 (3.1)	12 (4.6)	60 (22.9)	130 (49.8)	51 (19.6)	1	437 (96.6)	423 (96.79)

^a^Flies were fed three times per week (F3) on Monday, Wednesday and Friday; four times per week (F4) on Monday, Tuesday, Thursday and Friday; or six times per week (F6) from Monday to Saturday

^b^A, < 22 mg; B, 22 to < 28 mg; C, 28 to < 32 mg

^c^A quality factor of > 1 indicates that blood is suitable for colony maintenance

### Female productivity

On average, the first larva was deposited on day 15.75 in treatment F6, which was statistically similar to larval deposition in treatment F3 (17.75 days) and F4 (16 days; likelihood-ratio test:* χ*^2^ = 0.575,* df* = 2, *P* = 0.750).

Female productivity and fecundity were highest in treatment F4, followed by treatment F6 and F3 (Table 1). Model results showed that female productivity in treatments F6 and F4 were similar (Tukey post-hoc test value:* Z* = 0.1, *P* = 0.995), but both were significantly higher than in treatment F3 (Tukey post-hoc test value:* Z* = 6.363, *P* < 0.001).

Pupae produced by the three blood treatments were concentrated in size classes A and B, covering 95% of overall productivity, with 5% in size class C and no pupae in size classes D and E. No difference in productivity was observed between blood-feeding treatments for size class A pupae. For size class B pupae, significantly more pupae were produced in treatments F6 and F4 than in treatment F3 (Tukey post-hoc test value: Z = 7.020, *P* < 0.001). For size class C pupae, significantly more pupae were produced in treatment F6 than in treatments F3 and F4 (Tukey post-hoc test value: Z = 3.072, *P* = 0.005).

The QFs obtained for all blood-feeding frequencies were > 1, with the highest value for treatment F6 (1.59 ± 0.10) and the lowest for treatment F3 (1.18 ± 0.10; Table 1). Mixed model analyses showed that the QF obtained in treatment F3 was significantly lower than that obtained in treatments F6 and F4 (Tukey post-hoc test value:* Z* = 4.283, *P* < 0.001). No difference was observed between treatments F4 and F6 (Tukey post-hoc test value:* Z* = 1.532, *P* = 0.276).

Dissections at the end of the experimental period of 30 days showed that all females were inseminated (Table 1). The reproductive status of females fed three times a week was markedly different to that of females fed either four or six times a week. However, the latter two were similar. The percentage of empty uteri due to abortion was more than three times higher (18.3%) for treatment F3 than for treatments F4 and F6 (5.1% and 4.6%, respectively; Table 1). An overall delay in female productivity was observed for treatment F3 with 24.4% of females harboring recently ovulated eggs at day 30 compared to 8.1% and 3.1% for treatments F4 and F6, respectively. It led to a delay in larval instar distribution with 57.3% of females carrying larvae in their uteri at day 30 compared to 86.1% and 92.3% for treatments F4 and F6, respectively.

### Progeny emergence rate and flight ability

The emergence rates of adult flies were between 92 and 96% for all blood-feeding frequencies (Table 1). The best model retained blood-feeding frequencies as a significant explanatory variable (likelihood-ratio test:* χ*^2^ = 22.951,* df* = 2,* P* < 0.001). Emergence rates were significantly higher in treatments F4 and F6 than in treatment F3 (Tukey post-hoc test value:* Z* = 3.597, *P* < 0.001). No difference was observed between treatments F4 and F6 (Tukey post-hoc test value:* Z* = 0.896, *P* = 0.370).

The flight ability rate (i.e. the number of operational flies) was lowest in treatment F3 and highest in treatment F4 (84 and 96.7%, respectively) (Table 1). The best model retained blood-feeding frequencies as a significant explanatory variable (likelihood-ratio test: *χ*^2^ = 36.914,* df* = 2,* P* < 0.001). Flight ability rates were significantly higher in treatments F4 and F6 than in treatment F3 (Tukey post-hoc test value:* Z* = 4.338, *P* < 0.001). No difference was observed between treatments F4 and F6 (Tukey post-hoc test value: *Z* = 1.824, *P* = 0.068).

## Discussion

One of the main constraints when applying the SIT is the production of sufficient good-quality male flies for sterilization and release. Tsetse flies are obligatory hematophagous insects; therefore, the quality of the blood diet and the frequency of blood-feeding are two of the most important parameters for producing high-quality flies. The quality of the blood diet depends on external factors, such as the nutritional status of animals or blood contamination (bacteria or chemicals); consequently, the blood is always checked before being used in the insectary (i.e. blood quality control). Similarly, blood-feeding frequency can be optimized according to the requirements of the tsetse species and production parameters. In this study, we assessed the effect of different feeding frequencies on tsetse fly production parameters in a mass-rearing insectary.

Survival, fecundity and pupal size are the three essential comprehensive parameters of female tetse flies routinely used for assessing colony performance. In this study, the best results for all three parameters were obtained in treatment F4 (4 blood-feedings per week), with lower results obtained in treatment F6 (6 blood-feedings per week), although the differences were not significant. These results highlight that feeding flies four times per week instead of six times per week will have no adverse effect on colony production parameters. However, this was not the case for treatment F3 (3 blood-feedings per week); this feeding regimen led to significant negative effects on female survival and production parameters although fly maintenance remained acceptable. Increases in colony parameters between treatments F6 and F4 could be explained by the reduction in handling time for routine colony maintenance. Indeed, the transfer of fly cages to the feeding room involves some small and rapid changes in environmental conditions. Daily exposure to the 37 °C feeding membrane could be detrimental as temperatures above 30 °C may affect survival and fecundity [1, 26]. Although a feeding frequency of six times per week optimized the number of blood-fed flies, it is important to note that the tetse fly needs on average 2 days to completely digest a blood meal [27]. Therefore, daily feeding seems to be overoptimized from a physiological point of view, as well as unnecessary. This notion is supported by results of the QF values obtained for treatments F4 and F6. Both values were statistically similar, highlighting the fact that reducing the feeding frequency from 6 to 4 days per week has no effect on colony production. It should be noted that larger pupae were produced in treatment F6 than in treatments F3 and F4. While class C pupae represented only 9% of the F6 treatment production, it explains why the QF value for treatment F6 is slightly higher than that for F4 even though all production parameters were higher for treatment F4. Regarding the results of the progeny emergence rate and flight ability, reducing the feeding frequency to 4 days per week had no effect on progeny quality. However, a feeding frequency of 3 days per week led to a significant reduction in pupae emergence rate and flight ability. These results are probably the consequence of a lower acquisition of fat reserves in the larvae during the intrauterine phase, which is mainly dependent on the amount of blood acquired by the female during the interlarval period [28]. Although the results of treatment F3 are acceptable (89% emergence and 84% operational flies), on a colony-wide basis, there is an enormous difference in progeny quality in comparison to treatments F4 and F6. Similar findings were recently reported for *Glossina pallidipes*: flies fed five times per week showed better production parameters than when fed three times per week [29]. However, some tsetse species, such as *Glossina morsitans submorsitans*, seem to be more adapted to starvation. Indeed, in one study no differences were observed for the production parameters of these flies fed three, four or six times per week [30].

Female dissections showed that all flies were inseminated. Although the filling level of spermathecae in females was not mentioned, the results of this study indicate that feeding frequency did not influence the mating performance of males. However, a feeding frequency of 3 days per week led to an important increase in female abortion rate and an overall delay in pupae productivity. This feeding frequency is probably suboptimal and did not meet females’ physiological needs to produce pupae for a mass-rearing insectary.

From an economic standpoint, feeding frequency has an important impact on insectary running costs. Indeed, a high feeding frequency leads to higher personnel and infrastructure costs (water and electricity, which are especially expensive in Africa). As a result, such costs need to be balanced by an increase in pupae production. However, treatment F6 produced no more pupae than treatment F4 during the 30-day survey. Although the difference was not significant, it could be assumed that reducing feeding frequency from 6 days per week to 4 days per week will lead to significant cost savings while increasing or at least maintaining the same colony performance. At the same time, it would reduce the amount of blood used per week and therefore the frequency of blood collection at the slaughterhouse. It can be assumed that a reduction in feeding frequency as suggested here will lead to savings of approximately 30% per year. Reducing feeding to 3 days per week is also possible, but our results highlight that this blood-feeding frequency will have a significant effect on colony performance; as such, we do not recommend it for a mass-rearing insectary.

## Conclusions

In conclusion, the results of this study demonstrate that the feeding frequency of the CIRDES mass-rearing colony of *G. p. gambiensis* could be reduced to four times per week without affecting mass-rearing production and progeny quality. Moreover, it will have a positive economic impact that could enable more resources to be reinjected into the insectary.

## Data Availability

The data that support the findings of this study are openly available in Dataverse at http://dx.doi.org/10.18167/DVN1/1SPLYD
